# Association between HEI-2015 and hearing loss among American adults: National Health and Nutrition Examination Survey

**DOI:** 10.1017/S0022215125000635

**Published:** 2025-09

**Authors:** Juan Jiang, WanLei Chi

**Affiliations:** Tongde Hospital of Zhejiang Province, Hangzhou, China

**Keywords:** Healthy Eating Index, hearing loss, NHANES, noise exposure

## Abstract

**Objectives:**

The aim of this study was to investigate the association between the Healthy Eating Index 2015 scores and hearing loss.

**Methods:**

This study utilized cross-sectional data from individuals aged over 20 years (n = 5171) who participated in the National Health and Nutrition Examination Survey from 1999 to 2012 and 2015 to 2018. We collected information on their hearing, Healthy Eating Index 2015 scores and several other important covariates using multivariate regression analyses.

**Results:**

After adjusting for potential confounders, when hearing loss was defined as greater than or equal to 20 dB, the odds ratio for low-frequency hearing loss and high-frequency hearing loss was 0.99 (95 per cent confidence interval: 0.98–0.99; *p* < 0.001) and 0.99 (95 per cent confidence interval: 0.98–1; *p* = 0.006), respectively. When hearing loss was defined as greater than 25 dB, the odds ratio for low-frequency hearing loss and speech-frequency band hearing loss was 0.98 (95 per cent confidence interval: 0.98–0.99; *p* < 0.001) and 0.99 (95 per cent confidence interval: 0.98–1; *p* = 0.008), respectively.

**Conclusion:**

In American adults, Healthy Eating Index scores are associated with hearing loss.

## Introduction

The World Health Organization estimated that 466 million people, or 6.1 per cent of the global population, were living with disabling hearing loss (HL) in 2018, and this number is expected to increase as the population ages rapidly.^1^ In 2019, World Health Organization estimated the annual global cost of HL to be $750 billion. This not only causes considerable economic losses but also seriously reduces the quality of human life. HL has recently been ranked as the fifth leading cause of years lived with a disability.^2^ Many large epidemiological studies have found that HL is independently associated with falls, isolation, cognitive decline, dementia, anxiety, depression, social isolation, increased rates of hospitalization and healthcare use.^3^^–^^6^ This emphasizes the importance of early detection to potentially delay or prevent its onset. The pathogenesis of HL involves various factors, including microcirculation disorders, viral infections such as rubella and measles, head trauma, genetic factors, noise exposure, autoimmune diseases and so forth. These factors can lead to damage or degeneration of the hair cells in the cochlea, disruption of the auditory nerve pathways or impairment of the auditory processing in the brain, ultimately resulting in a decrease in hearing function.^7^ Multiple studies have shown that the dietary intake of carbohydrates, cholesterol, fiber, protein, sugar, fruits, vegetables, saturated fats and trans-fats is associated with self-reported HL and requires public health interventions to prevent HL^8^^–^^11^; however, comprehensive guidelines on healthy eating are lacking. As the concept of dietary patterns continues to develop and evolve, the Healthy Eating Index (HEI) is widely used in different types of research, including surveillance, epidemiological and intervention studies, involving different populations in the United States. It can be indexed to guide the diet of Americans for early prevention of HL.^12^ The HEI is a measure that assesses whether a group of foods meets the Dietary Guidelines for Americans, which measures diet quality rather than quantity; that is, it assesses density rather than absolute quantity, providing a guide to the overall diet.^13^ There were no changes in the components or standards between HEI-2015 and HEI-2020.^14^ The relation between the HEI and HL is an important topic in healthcare and public health. In this context, by conducting a retrospective cross-sectional study involving 5,171 U.S. adults from the National Health and Nutrition Examination Survey (NHANES), we sought to clarify the relation between the HEI and HL in adults.

### Materials and methods

**Figure 1.**
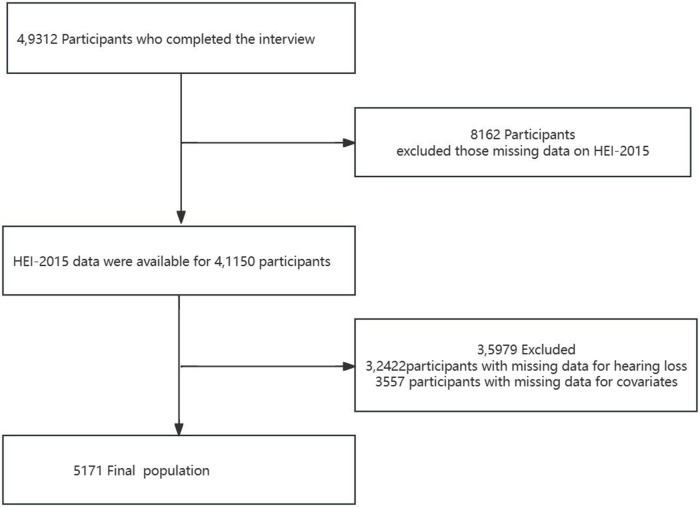
The study’s flow diagram.

This cross-sectional study utilized NHANES data from 1999 to 2012 and 2015 to 2018, conducted by the Centers for Disease Control and Prevention.^15^ The purpose of the NHANES was to assess the health and nutritional status of non-hospitalized Americans. NHANES collects demographic and in-depth health information through home visits, screenings and laboratory tests at mobile screening centers. The NHANES was authorized by the National Center for Health Statistics Ethics Review Committee, and all participants provided written informed consent prior to participation. The secondary analysis did not require additional approval from the Institutional Review Board.^16^ Data from NHANES may be obtained through the NHANES website (visited on March 1, 2022; http://www.cdc.gov/nchs/nhanes.htm). Individuals aged over 20 years who completed the interviews participated in our study. We excluded individuals who lacked data on HL, HEI, or covariates. The total sample size of adults evaluated was N = 49,312, the details of which are described in Figure 1. Only publicly available data were used in the analysis, and ethical approval was not required for this study.

The HEI assesses diet quality and refers to how well a group of foods meets the Dietary Guidelines for Americans.^17^ HEI-2020 contains 13 components and scoring criteria, and this is the first time that there has been no change in the index; the score of HEI-2020 is the same as that of HEI-2015. Krebs-Smith *et al*. described the details of each component in the HEI-2015 update.^13^ The HEI-2015 score ranges from 0 to 100 and is graded as follows: Grade A (highest score): Total score between 90 and 100 points, indicating that an individual’s dietary nutrient intake is very consistent with dietary guidelines. Grade B: Total score between 80 and 89 points, indicating that an individual’s dietary nutrient intake is somewhat consistent with dietary guidelines. Grade C: Total score between 60 and 79 points, indicating that an individual’s dietary nutrient intake has room for improvement, but can still adopt a healthy eating pattern. Grade D: Total score below 60 points, indicating that an individual’s dietary nutrient intake needs significant adjustment to improve dietary quality. Grade F: Total score between 0 and 59 points, indicating that an individual’s dietary nutrient intake is severely inadequate and requires special attention and improvement. A higher score reflects better diet quality. We used Day 1 Total Nutrient Intake to calculate the 13 components of HEI-2015.

All hearing tests were performed by a trained examiner on candidates aged 20–69 years in a dedicated soundproof room at a mobile test center. Hearing thresholds were tested in both ears at seven frequencies (500, 1000, 2000, 3000, 4000, 6000 and 8000 Hz), with observed values ranging between -10 and 120 dB. The pure tone average (PTA) of speech-frequency in both ears was calculated as the average of the hearing thresholds at 0.5, 1, 2 and 4 kHz. Good ear PTA is a continuous variable; the higher the value, the worse the hearing. Low-frequency PTA calculations used hearing thresholds at 0.5, 1 and 2 kHz in the better ears, while high-frequency PTA calculations used hearing thresholds at 4, 6 and 8 kHz in the better ears. All hearing thresholds were reported as dB HL. Sensitivity analysis was performed using PTA with the bad ear rather than the good ear. In addition, good ear PTAs were classified according to clinical cutoff points defined by the 1997 World Health Organization, where a hearing level less than or equal to 25 dB HL indicates normal hearing and >25 dB HL indicates HL.^18^ As defined by the World Health Organization in 2021, HL is defined as the speech-frequency of good ear PTA greater than or equal to 20 dB, and less than 20 dB is normal hearing.^19^

Multiple potential covariates were evaluated based on literature,^20^^–^^22^ including age, sex, marital status, race/ethnicity, education level, household income, smoking status, physical activity, high blood pressure, diabetes mellitus (DM), history of cardiovascular disease (CVD), body mass index (BMI) and noise exposure. Race/ethnicity was classified as Mexican American, other Hispanic, non-Hispanic White, non-Hispanic Black or other races, including multiple races. Marital status was divided into married, never married, living with a partner, widowed, divorced or separated. The level of education was classified as less than 9th grade, 9–11th grade (including 12th grade with no diploma), high school graduate/General Educational Development or equivalent, college or Associate of Arts (AA) degree and college graduate or above. Smoking status, as defined in previous literature, was classified as never smoked (smoking less than 100 cigarettes), current smoker or former smoker (quitting after smoking more than 100 cigarettes). Physical activity was classified as sedentary, moderate (at least 10 min of exercise in the past 30 days that resulted in only slight sweating or a mild-to-moderate increase in breathing or heart rate) and vigorous (at least 10 min of exercise in the past 30 days that resulted in heavy sweating or an increase in breathing or heart rate). Previous medical conditions (including hypertension, DM, stroke and coronary heart disease) were determined based on questions in the questionnaire regarding whether the doctor had been informed of the condition in the past. BMI was calculated using a standardized technique based on weight and height. Noise exposure included exposure to gun noise outside the workplace, noise outside the workplace, working in a noisy environment and exposure to loud noise at work.

For statistical analysis, categorical variables were expressed as proportions (per centages), and continuous variables were expressed as mean (standard deviation) or median (interquartile distance). To compare the differences between groups, one-way analysis of variance (ANOVA) (normal distribution), Kruskal–Wallis test (skewed distribution) and chi-squared test (categorical variables) were performed. After adjusting for all covariates, linear regression was used to describe the relation between the healthy diet index and hearing. Logistic regression models were used to determine the odds ratios (ORs) and 95 per cent confidence intervals (CIs) between HEI and HL. Model 1 was adjusted for sociodemographic characteristics, including age, sex, race, marital status, household income and educational level. Model 2 was adjusted for sociodemographic characteristics, smoking status, alcohol consumption, physical activity and BMI. Model 3 was comprehensively adjusted for sociodemographic characteristics, smoking status, alcohol consumption, physical activity, BMI, CVD, hypertension, diabetes mellitus (DM) and noise exposure.

Furthermore, potential changes in the relation between healthy dietary index and HL were assessed, including the following variables: age (20–65 years vs >65 years), sex, marital status (married vs. never married, living with a partner or other [widowed, divorced or separated individuals]) and BMI (<25 vs. 25–30, ≥30 kg/m^2^). Multivariate logistic regression was used to assess the heterogeneity among the subgroups, and the interaction between the subgroups and the healthy diet index was examined using the likelihood ratio test.

All analyses were performed using the statistical package R 4.3.1 (http://www.R-project.org; R Foundation, Shanghai, China) (accessed on March 10, 2024) and Free Statistics software version 1.9. Descriptive statistics were calculated for all participants. In the bilateral test, statistical significance was set at *p* less than 0.05.

## Results

### Study population

A total of 49,312 participants, all aged 20 years and older, completed interviews. We excluded those with missing data on the HEI-2015 (*n* = 8,162), those with missing data on HL (*n* = 32,422) or those with covariates (*n* = 3,557). Ultimately, this cross-sectional study includes 5,171 participants from the NHANES between 1999 and 2012, and 2015 and 2018. The detailed inclusion and exclusion processes are presented in Figure 1.

### Baseline characteristics

Table 1 illustrates the baseline characteristics of all participants according to the HEI-2015. We included a total 5,171 patients with a mean age of 50.9 plus or minus 18.5 years, among whom 60.9 per cent were non-Hispanic White and 52.9 per cent were men. HL was defined according to speech-frequency PTA in the better-hearing ear, with greater than 25 dB HL and greater than or equal to 20 dB HL as having HL. The overall prevalence rates of HL in the study population were 25.0 per cent and 37.6 per cent, respectively.

**Table 1.** Population characteristics by categories of the HEI-2015

Characteristic	HEI−2015	
	No.	*p*-Value
Age, mean ± SD	50.9 ± 18.5	< 0.001
Sex, n (%)		< 0.001
Male	2736 (52.9)	
Female	2435 (47.1)	
Race, n (%)		< 0.001
Mexican American	833 (16.1)	
Other Hispanic	174 (3.4)	
Non-Hispanic White	3151 (60.9)	
Non-Hispanic Black	851 (16.5)	
Other race-including multi-racial	162 (3.1)	
Marital status, n (%)		< 0.001
Married	3048 (58.9)	
Never married	761 (14.7)	
Living with partner	317 (6.1)	
Other: widowed, divorced, or separated individuals	1045 (20.2)	
PIR, mean ± SD	2.9 ± 1.6	< 0.001
Education, n (%)		< 0.001
Less than 9th grade	455 (8.8)	
9−11th grade (includes 12th grade with no diploma)	637 (12.3)	
High school grad/GED or equivalent	1287 (24.9)	
Some college or AA degree	1563 (30.2)	
College graduate or above	1229 (23.8)	
Smoker, n (%)		< 0.001
Never	2540 (49.1)	
Former	1537 (29.7)	
Now	1094 (21.2)	
Alcohol consumption, n (%)		< 0.001
Never	625 (12.1)	
Former	965 (18.7)	
Mild	1887 (36.5)	
Moderate	792 (15.3)	
Heavy	902 (17.4)	
BMI_kg.m^2^, mean ± SD	28.2 ± 6.1	< 0.001
CVD, n (%)	574 (11.1)	< 0.001
Hypertension, n (%)	2177 (42.1)	< 0.001
DM, n (%)	682 (13.2)	< 0.001
Noise, n (%)	1708 (33.0)	0.04
Low-frequency hearing loss (≥20dB), n (%)	1112 (21.5)	< 0.001
Low-frequency hearing loss (>25 dB), n (%)	606 (11.7)	< 0.001
Speech-frequency hearing loss (≥20dB), n (%)	1946 (37.6)	< 0.001
Speech-frequency hearing loss (>25 dB) n (%)	1295 (25.0)	< 0.001
High-frequency hearing loss (≥20 dB) trans, n (%)	2674 (51.7)	< 0.001
High-frequency hearing loss (>25 dB), n (%)	2149 (41.6)	< 0.001
Physical activity, median (IQR)	480.0 (157.5, 1145.3)	< 0.001
Low-frequency hearing, median (IQR)	10.0 (5.0, 16.7)	< 0.001
Speech-frequency hearing, median (IQR)	13.8 (7.5, 26.2)	< 0.001
High-frequency hearing, median (IQR)	20.0 (10.0, 45.0)	< 0.001

BMI = body mass index; CVD = cardiovascular disease; DM = diabetes mellitus; GED = General Educational Development; HEI-2015 = Healthy Eating Index 2015; IQR = interquartile range; PIR = poverty income ratio.

The group with a higher HEI was observed to have a higher proportion of men; be non-Hispanic White and married; have a higher family poverty income ratio and educational level; be never smokers and mild alcohol users; have a lower BMI, greater physical activity, a lower incidence of stroke and history of CVD, no hypertension, no DM, no noise and normal hearing.

### Relation between the HEI-2015 and HL

Univariate analysis demonstrated that age, sex, race, marital status, family income, educational level, smoking status, alcohol consumption, physical activity, BMI, coronary heart disease, hypertension, DM and noise exposure were associated with low-frequency HL, speech-frequency HL and high-frequency HL (see Supplementary Material Tables 4, 5 and 6, respectively). In linear multifactor analysis (Table 2), after adjusting for potentially confounding factors, there was a significant negative association between the HEI-2015 and low-frequency hearing, with a coefficient of -0.03 (95 per cent CI: -0.05 to -0.01; *p* = 0.001); speech-frequency hearing, with a coefficient of -0.04 (95 per cent CI: -0.06 to -0.01; *p* = 0.003); and high-frequency hearing, with a coefficient of -0.05 (95% CI: -0.08 to -0.02; *p* = 0.003). When the HEI-2015 was a continuous variable, after adjusting for potential confounders, there was a significant negative association between the HEI-2015 and low-frequency HL (Table 3), with OR values of 0.99 (95 per cent CI: 0.98–0.99; *p* < 0.001) and 0.98 (95 per cent CI: 0.98–0.99; *p* < 0.001). The OR was 0.99 in speech-frequency HL, defined as HL greater than 25 dB (95 per cent CI: 0.98–1; *p* = 0.008), and the OR was 0.99 (95 per cent CI: 0.98–1; *p* = 0.006) in high-frequency HL, defined as HL greater than or equal to 20 dB. When the HEI-2015 was analyzed using quintiles (see Supplementary Material Table 7), a significant negative association was found between the HEI-2015 and HL after adjusting for potential confounders. The adjusted OR values of HEI-2015 and F-scores (0–59), D(60–69), C(70–79), B(80–89) and A(90–100) for HL are shown in Supplementary Material Table 7. With hearing less than 20 dB as the normal value, the OR value of low-frequency hearing in B(80–89) was 0.54 (95 per cent CI: 0.34–0.88; *p* = 0.013). The OR values of speech-frequency band listening were 0.7 (95 per cent v CI: 0.53–0.93; *p* = 0.013) in C(70–79) and 0.14 (95% CI: 0.03–0.56; *p* = 0.005) in A(90–100). The OR values for high-frequency hearing were 0.76 (95 per cent CI: 0.61–0.96; *p* = 0.02) in D(60–69) and 0.66 (95 per cent CI: 0.47–0.91; *p* = 0.012) in C(70–79), and the OR value in B(80–89) was 0.52 (95 per cent CI: 0.3–0.91; *p* = 0.021). However, when hearing was less than or equal to 25 decibels, the OR value of low-frequency hearing was 0.7 (95 per cent CI: 0.51–0.96; *p* = 0.029) in C(70–79) and 0.54 (95 per cent CI: 0.3–0.96; *p* = 0.035) in B(80–89).

**Table 2.** Association between the HEI-2015 and hearing

	Coefficient (95% CI)			
Variable	Crude	*p*-Value	Adjusted	*p*-Value
Low-frequency hearing	0.12 (0.1∼0.14)	<0.001	−0.03 (−0.05∼−0.01)	0.001
Speech-frequency hearing	0.17 (0.14∼0.2)	<0.001	−0.04 (−0.06∼−0.01)	0.003
High-frequency hearing	0.35 (0.3∼0.39)	<0.001	−0.05 (−0.08∼−0.02)	0.003

CI = confidence interval; HEI-2015 = Healthy Eating Index 2015.

**Table 3.** Association between the HEI-2015 and hearing loss

	OR (95% CI)								
Variable	No.	Crude	*p*-Value	Model 1	*p*-Value	Model 2	*p*-Value	Model 3	*p*-Value
Low-frequency hearing loss(≥20 dB)	1112	1.02 (1.02∼1.03)	<0.001	0.98 (0.98∼0.99)	<0.001	0.99 (0.98∼0.99)	<0.001	0.99 (0.98∼0.99)	<0.001
Low-frequency hearing loss (>25 dB)	606	1.02 (1.01∼1.03)	<0.001	0.98 (0.98∼0.99)	<0.001	0.98 (0.98∼0.99)	<0.001	0.98 (0.98∼0.99)	<0.001
Speech-frequency hearing loss (≥20 dB)	1946	1.03 (1.02∼1.03)	<0.001	0.99 (0.99∼1)	0.008	0.99 (0.99∼1)	0.079	0.99 (0.99∼1)	0.077
Speech-frequency hearing loss (>25 dB)	1295	1.02 (1.02∼1.03)	<0.001	0.99 (0.98∼1)	0.001	0.99 (0.98∼1)	0.008	0.99 (0.98∼1)	0.008
High-frequency hearing (≥20 dB)	2674	1.03 (1.02∼1.03)	<0.001	0.99 (0.98∼0.99)	0.001	0.99 (0.98∼1)	0.008	0.99 (0.98∼1)	0.006
High-frequency hearing (>25 dB)	2149	1.03 (1.03∼1.04)	<0.001	0.99 (0.99∼1)	0.097	1 (0.99∼1)	0.343	1 (0.99∼1)	0.307

BMI = body mass index; CI = confidence interval; CVD = cardiovascular disease; DM = diabetes mellitus; OR = odds ratio; PIR = poverty income ratio; Ref: reference.

Model 1 was adjusted for sociodemographic variables (age, sex, race, marital status, PIR, education). Model 2 was adjusted for sociodemographic variables (age, sex, race, marital status, PIR, education), smoking status, alcohol, physical activity, BMI. Model 3 was adjusted for sociodemographic variables (age, sex, race, marital status, PIR, education), smoking status, alcohol consumption, physical activity, BMI, CVD, hypertension, DM, noise.

### Stratified analyses based on additional variables

Stratified analysis was performed on several subgroups to assess the potential impact of the relation between the HEI-2015 and HL. When stratified by sex, age, BMI and marital status, no significant interactions were observed between the subgroups. Given the sample size, a *p*-value of less than 0.05 for marriage and BMI may not be statistically significant (see Supplementary Material Figures 2, 3 and 4).

## Discussion

We found that the HEI scores were associated with HL, and subgroup analyses showed stable results.

Spankovich’s findings support the link between healthier diets and lower high-frequency thresholds in adults.^23^ Adherence to healthy eating patterns has been linked to a reduced risk of HL in women.^24^ An Australian cross-sectional study found that the overall diet quality was associated with concurrent vision and HL.^25^ Previous studies have linked a healthy diet to HL, which is similar to our findings.

Some studies have suggested that dietary supplement use is positively associated with hearing improvement at all frequencies.^26^ Studies by Rosenhall *et al*. have shown that fish are beneficial for hearing, while eating “junk food” rich in low-molecular-weight carbohydrates is detrimental.^27^ A diet high in saturated fat, cholesterol and carbohydrates is a risk factor for HL, and conversely, increasing antioxidants in the form of protein, zinc, magnesium, selenium, iron, iodine, fruits, vegetables, polyunsaturated fatty acids (omega-3) and vitamins A, C and E can prevent HL from developing.^8^^,^^28^^–^^30^ Ji Eun Choi *et al*. believe that a high intake of seeds, nuts, fruits, seaweed and vitamin A has protective effects on hearing, and dietary antioxidants or anti-inflammatory foods may help reduce the occurrence of HL.^31^ Xinmin Wei believes that the dietary intake of magnesium and calcium is associated with a lower risk of HL.^32^ Many scholars have studied the relation between diet and HL; some diets are protective factors, whereas others are risk factors.

Most previous studies on diet and hearing have focused on the analysis of single nutrients, with the limitation that nutrient intake is correlated, making it difficult to isolate the effects of one nutrient from those of others. Single-nutrient analyses, which do not consider biochemical interactions between nutrients, also increase the possibility of false-positive associations.^33^ As a supplement to single-nutrient analysis, dietary pattern analysis can better capture the synergistic and cumulative effects of total dietary intake on health outcomes.^34^

The HEI assesses whether a group of foods meets the American Dietary Guidelines for Americans. The HEI can be applied to any group of foods by assigning a score to each component and comparing its density to the relevant criteria. HEI scores have been widely used in numerous studies on diet quality in populations, the relation between diet quality and health outcomes, the impact of interventions on diet quality and economic and food-context-based studies.^13^ The HEI includes eating more fruits, vegetables, legumes, whole grains, nuts, dairy products, seafood, plant proteins and fatty acids, and less saturated fat, sugar and sodium. There are many mechanisms by which this healthy eating pattern prevents HL, including the prevention of microvascular and macrovascular damage to cochlear blood flow, inhibition of oxidative damage and reduction of inflammation.^24^ A lower high-fat diet in a high-quality diet may reduce induced oxidative stress, mitochondrial damage and apoptosis in the inner ear, which has a protective effect on inner ear cells.^35^^,^^36^ Inadequate blood supply to the cochlea can lead to hypoxia and ischemic injury, oxidative stress, mitochondrial dysfunction, cellular damage and peripheral and central auditory neurodegeneration.^37^ Dietary nutrients of polyunsaturated fatty acids and multivitamins, such as vitamins B12 and C, calcium and selenium, may have antioxidant and neuroprotective effects.^38^ They may also protect against neurodegeneration of auditory nerve fibers and central auditory pathways.^24^ Dietary guidance for patients with potential HL is provided by examining the effects of a healthy diet index on HL.

This study has several limitations. First, the cross-sectional design was a major limitation, and no causal relation could be inferred from this study. Second, the use of self-reported 24-hour food recall data is limited because they are prone to overestimation or underestimation. Finally, more participants were excluded because of the lack of data on any covariates that could have affected the results. Despite these limitations, our study has several strengths. The use of a large, nationally representative database to assess diet quality is a major strength of the present study.

We found an association between the HEI-2015 score and HL. Therefore, attention should be paid to the relation between dietary quality and HL. Future cohort studies or randomized controlled trials are needed to confirm this relation.

What is already known

- Hearing loss (HL) is a major global health burden linked to cognitive decline, depression and reduced quality of life, with modifiable risk factors understudied
- Prior studies suggest associations between single nutrients (e.g., vitamins, fatty acids) and HL, but evidence on overall dietary patterns remains limited
- The Healthy Eating Index (HEI) is a validated tool for assessing diet quality, yet its relationship with HL in adults is poorly characterized

What this paper adds

- First large-scale study demonstrating a significant inverse association between HEI-2015 scores and HL across low-, speech- and high-frequency thresholds in U.S. adults
- Higher HEI-2015 scores (indicating better diet quality) correlate with reduced odds of HL, particularly in quintile-based analyses (e.g., OR = 0.52 for high-frequency HL in the 80–89 score group)
- Provides actionable evidence for integrating dietary guidelines into HL prevention strategies, highlighting diet quality as a modifiable lifestyle factor
- Strengthens the rationale for future longitudinal studies to explore causal links between diet and auditory health

## Conclusions

We found that higher HEI scores were associated with better hearing and that a healthy diet may also help reduce the risk of HL.

## Supporting information

Jiang and Chi supplementary material 1 Jiang and Chi supplementary material

Jiang and Chi supplementary material 2 Jiang and Chi supplementary material

## Supplementary material

The supplementary material for this article can be found at https://doi.org/10.1017/S0022215125000635.

## References

[1] GBD 2017 Disease and Injury Incidence and Prevalence Collaborators. Global, regional, and national incidence, prevalence, and years lived with disability for 354 diseases and injuries for 195 countries and territories, 1990-2017: a systematic analysis for the Global Burden of Disease Study 2017. *Lancet* 2018;392:1789–858 30496104 10.1016/S0140-6736(18)32279-7 PMC6227754

[2] Stika CJ, Hays RD. Development and psychometric evaluation of a health-related quality of life instrument for individuals with adult-onset hearing loss. *Int J Audiol* 2015;55:381–91 27104754 10.3109/14992027.2016.1166397 PMC5005284

[3] Jiam NT, Li C, Agrawal Y. Hearing loss and falls: a systematic review and meta-analysis. *Laryngoscope* 2016;126: 2587–96 27010669 10.1002/lary.25927

[4] Kirkpatrick SI, Reedy J, Krebs-Smith SM, Pannucci TE, Subar AF, Wilson MM, et al. Applications of the Healthy Eating Index for surveillance, epidemiology, and intervention research: considerations and caveats. *J Acad Nutr Diet* 2018;118:1603–21 30146072 10.1016/j.jand.2018.05.020 PMC6730554

[5] Slade K, Plack CJ, Nuttall HE. The effects of age-related hearing loss on the brain and cognitive function. *Trends Neurosci* 2020;43:810–21 32826080 10.1016/j.tins.2020.07.005

[6] Blazer DG, Tucci DL. Hearing loss and psychiatric disorders: a review. *Psychol Med* 2019;49:891–7 30457063 10.1017/S0033291718003409

[7] Chandrasekhar SS, Tsai Do BS, Schwartz SR, Bontempo LJ, Faucett EA, Finestone SA, et al. Clinical practice guideline: sudden hearing loss (update). *Otolaryngol Head Neck Surg* 2019;161:S1–45 31369359 10.1177/0194599819859885

[8] Rodrigo L, Campos-Asensio C, Rodríguez MÁ, Crespo I, Olmedillas H. Role of nutrition in the development and prevention of age-related hearing loss: a scoping review. *J Formos Med Assoc* 2021;120:107–20 32473863 10.1016/j.jfma.2020.05.011

[9] Dillard LK, Nelson-Bakkum E, Schultz A, Merten N, Malecki K. Associations of dietary intake with self-reported hearing loss: findings from the survey of the Health of Wisconsin. *J Speech Lang Hear Res* 2023;66:2478–89 37263020 10.1044/2023_JSLHR-22-00473

[10] Zhang X, Luo Q, Huang Z, Xiang X. Association between nineteen dietary fatty acids and hearing thresholds: findings from a nationwide survey. *Lipids Health Dis* 2023;22:126 37563575 10.1186/s12944-023-01896-y PMC10413493

[11] Chen H-L, Tan C-T, Wu C-C, Liu T-C. Effects of diet and lifestyle on audio-vestibular dysfunction in the elderly: a literature review. *Nutrients* 2022;14:4720 36432406 10.3390/nu14224720 PMC9698578

[12] Schap TR, Kuczynski K, Hiza H. Healthy Eating Index—Beyond the Score. *J Acad Nutr Diet* 2017;117:519–21 28343522 10.1016/j.jand.2017.02.002

[13] Krebs-Smith SM, Pannucci TRE, Subar AF, Kirkpatrick SI, Lerman JL, Tooze JA, et al. Update of the Healthy Eating Index: HEI-2015. *J Acad Nutr Diet* 2018;118:1591–602 30146071 10.1016/j.jand.2018.05.021 PMC6719291

[14] Shams-White MM, Pannucci TRE, Lerman JL, Herrick KA, Zimmer M, Mathieu KM, et al. Healthy Eating Index-2020: review and update process to reflect the dietary guidelines for Americans, 2020-2025. *J Acad Nutr Diet* 2023;123:1280–8 37201748 10.1016/j.jand.2023.05.015 PMC10524328

[15] National Center for Health Statistics. NHANES Survey Methods and Analytic Guidelines. In: https://wwwn.cdc.gov/nchs/nhanes/analyticguidelines.aspx [1 June 2025]

[16] US Department of Health & Human Services. National Institutes of Health, Office of Extramural Research, NIH Grants Policy Statement. Bethesda, MD: OER, NIH; 2015. Available from: https://grants.nih.gov/policy/nihgps/index.htm [1 March 2022]

[17] Nutrition and Your Health: 2015–2020 Dietary Guidelines for Americans, 8th edn. Washington, DC: US Government Printing Office, 2015. https://odphp.health.gov/sites/default/files/2019-09/2015-2020_Dietary_Guidelines.pdf

[18] Informal Working Group on Prevention of Deafness and Hearing Impairment Programme Planning (1991: Geneva, Switzerland) & World Health Organization. Programme for the Prevention of Deafness and Hearing Impairment. (1991). Report of the Informal Working Group on Prevention of Deafness and Hearing Impairment Programme Planning, Geneva, 18-21 June 1991. In: https://iris.who.int/handle/10665/58839

[19] Jiang K, Spira AP, Reed NS, Lin FR, Deal JA. Sleep characteristics and hearing loss in older adults: The National Health and Nutrition Examination Survey 2005-2006. *J Gerontol A Biol Sci Med Sci* 2022;77:632–9 34302481 10.1093/gerona/glab214 PMC9122752

[20] Jung W, Kim J, Cho IY, Jeon KH, Song Y-M. Association between serum lipid levels and sensorineural hearing loss in Korean adult population. *Korean J Fam Med* 2022;43:334–43 36168906 10.4082/kjfm.21.0148 PMC9532192

[21] Zou P, Li M, Chen W, Ji J, Xue F, Wang Z, et al. Association between trace metals exposure and hearing loss. *Front Public Health* 2022;10:973832 36062090 10.3389/fpubh.2022.973832 PMC9428401

[22] Scinicariello, F, Przybyla J, Carroll Y, Eichwald J, Decker J, Bresse PN. Age and sex differences in hearing loss association with depressive symptoms: analyses of NHANES 2011-2012. *Psychol Med* 2019;49:962–8 29909806 10.1017/S0033291718001617 PMC8114788

[23] Spankovich C, Le Prell CG. Healthy diets, healthy hearing: National Health and Nutrition Examination Survey, 1999-2002. *Int J Audiol* 2013;52:369–76 23594420 10.3109/14992027.2013.780133 PMC4036465

[24] Curhan SG, Wang M, Eavey RD, Stampfer MJ, Curhan GC. Adherence to healthful dietary patterns is associated with lower risk of hearing loss in women. *J Nutr* 2018;148:944–51 29757402 10.1093/jn/nxy058 PMC6481387

[25] Gopinath B, Schneider J, Flood VM, McMahon CM, Burlutsky G, Leeder SR, et al. Association between diet quality with concurrent vision and hearing impairment in older adults. *J Nutr Health Aging* 2014;18:251–624626751 10.1007/s12603-013-0408-x

[26] Kang JW, Choi HS, Kim K, Choi JY. Dietary vitamin intake correlates with hearing thresholds in the older population: the Korean National Health and Nutrition Examination Survey. *Am J Clin Nutr* 2014;99:1407–13 24646817 10.3945/ajcn.113.072793

[27] Rosenhall U, Idrizbegovic E, Hederstierna C, Rothenberg E. Dietary habits and hearing. *Int J Audiol* 2015;54:S53–6 25549171 10.3109/14992027.2014.972524

[28] Kim SY, Sim S, Kim H-J, Choi HG. Low-fat and low-protein diets are associated with hearing discomfort among the elderly of Korea. *Br J Nutr* 2015;114:1711–17 26388267 10.1017/S0007114515003463

[29] Zhang X, Luo Q, Huang Z, Xiang X. Association between nineteen dietary fatty acids and hearing thresholds: findings from a nationwide survey. *Lipids Health Dis* 2023;22:126 37563575 10.1186/s12944-023-01896-y PMC10413493

[30] Jung SY, Kim SH, Yeo SG. Association of nutritional factors with hearing loss. *Nutrients* 2019;11:307 30717210 10.3390/nu11020307 PMC6412883

[31] Choi, JE, Ahn J, Moon IJ. Associations between age-related hearing loss and dietary assessment using data from Korean National Health and Nutrition Examination Survey. *Nutrients* 2021;13:1230 33917838 10.3390/nu13041230 PMC8068238

[32] Wei, X. Dietary magnesium and calcium intake is associated with lower risk of hearing loss in older adults: a cross-sectional study of NHANES. *Front Nutr* 2023;10:1101764 36998904 10.3389/fnut.2023.1101764 PMC10043168

[33] Dawes, P, Cruickshanks, KJ, Marsden, A, Moore, DR, Munro, KJ. Relationship between diet, tinnitus, and hearing difficulties. *Ear Hear* 2020;41:289–99 31356390 10.1097/AUD.0000000000000765 PMC7664714

[34] McGuire S. Scientific report of the 2015 Dietary Guidelines Advisory Committee. Washington, DC: US Departments of Agriculture and Health and Human Services, 2015. *Adv Nutr* 2016;7:202–4 10.3945/an.115.011684 PMC4717899 26773024

[35] Tang T-H, Hwang J-H, Yang T-H, Hsu C-J, Wu C-C, Liur T-C. Can nutritional intervention for obesity and comorbidities slow down age-related hearing impairment? *Nutrients* 2019;11:1668 31330876 10.3390/nu11071668 PMC6682960

[36] Le Prell CG, Gagnon PM, Bennett DC, Ohlemiller KK. Nutrient-enhanced diet reduces noise-induced damage to the inner ear and hearing loss. *Transl Res* 2011;158:38–53 21708355 10.1016/j.trsl.2011.02.006 PMC3132794

[37] Yamasoba Y, Lin FR, Someya S, Kashio A, Sakamoto T, Kondo K.. Current concepts in age-related hearing loss: epidemiology and mechanistic pathways. *Hear Res* 2013;303:30–8 23422312 10.1016/j.heares.2013.01.021 PMC3723756

[38] El Soury M, Fornasari BE, Carta, G, Zen, F, Haastert-Talini, K, Ronchi, G. et al. The role of dietary nutrients in peripheral nerve regeneration. *Int J Mol Sci* 2021;22:7417 34299037 10.3390/ijms22147417 PMC8303934

