# Supplementary material for: Association between HEI-2015 and hearing loss among American adults: National Health and Nutrition Examination Survey
Source: J Laryngol Otol. 2025 Sep;139(9):881–7. doi: 10.1017/S0022215125000635 (PMC12571590; doi:10.1017/S0022215125000635)

Figure 2 and Figure 3, Figure 4

Figure2. The relationship between low-frequency hearing loss and the Healthy Eating Index-15(HEI-2015) from basic characteristics. In addition to the layered components themselves, Each stratified factor was adjusted for all other variables (Race, PIR, Education, smoke, alcohol, physical activity, CVD, hypertension, DM, noise) , LFHL:low-frequency hearing loss.


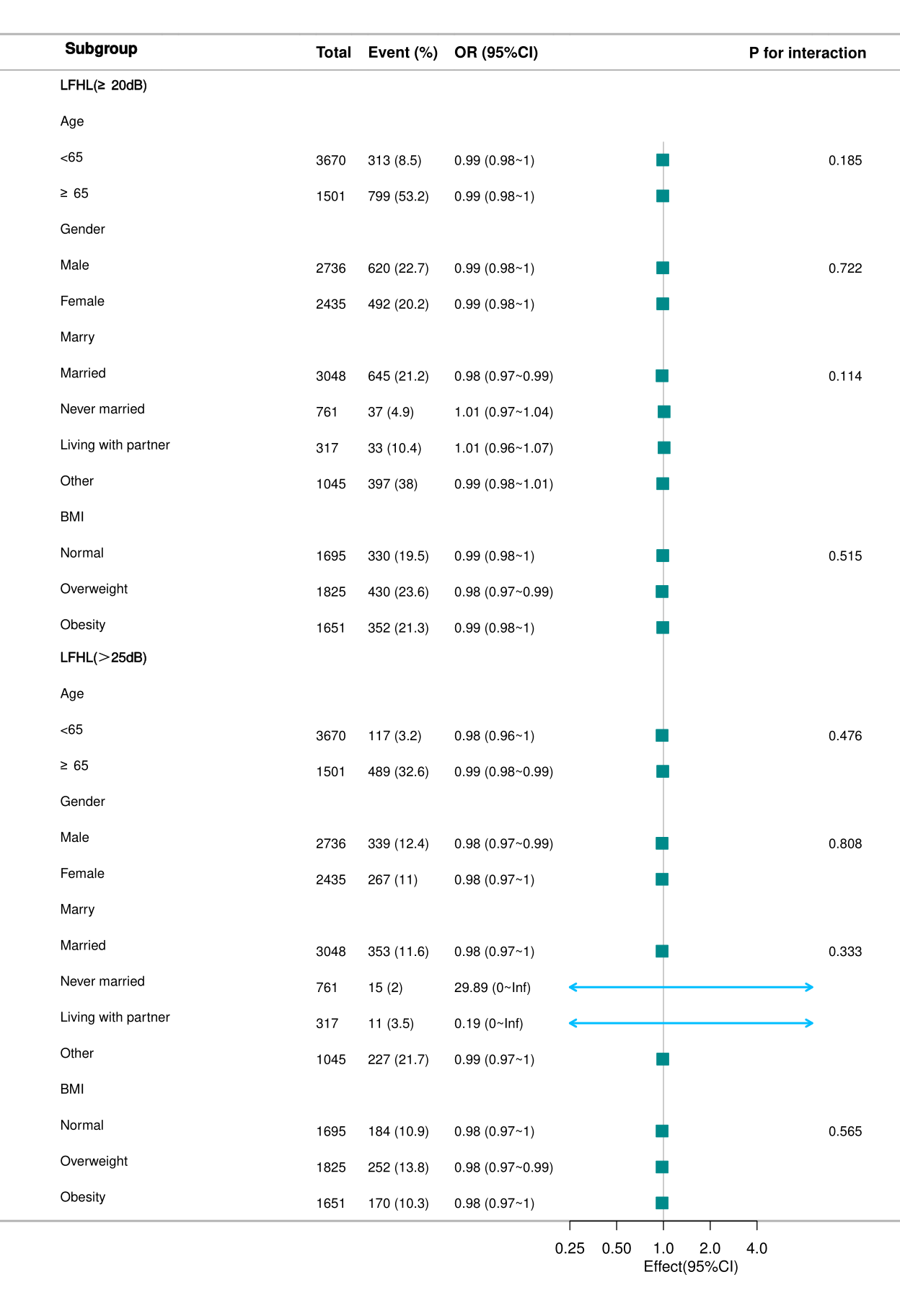


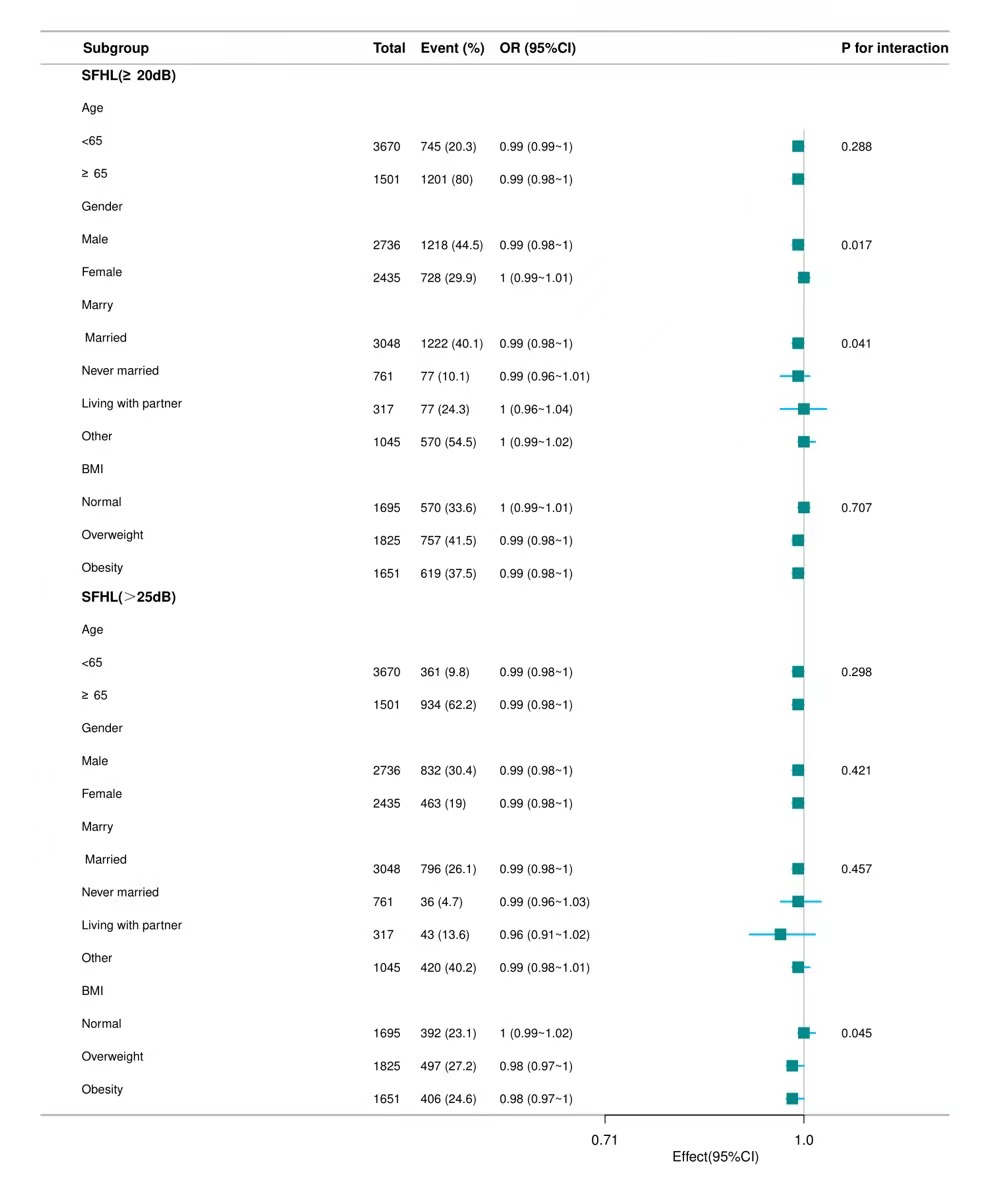


Figure3. The relationship between speech-frequency hearing loss and the Healthy Eating Index-15(HEI-2015) from basic characteristics. In addition to the layered components themselves, Each stratified factor was adjusted for all other variables (Race, PIR, Education, smoke, alcohol, physical activity, CVD, hypertension, DM, noise), SFHL:speech-frequency hearing loss.

Figure4. The relationship between speech-frequency hearing loss and the Healthy Eating Index-15(HEI-2015) from basic characteristics. In addition to the layered components themselves, Each stratified factor was adjusted for all other variables (Race, PIR, Education, smoke, alcohol, physical activity, CVD, hypertension, DM, noise), HFHL:high-frequency hearing loss.


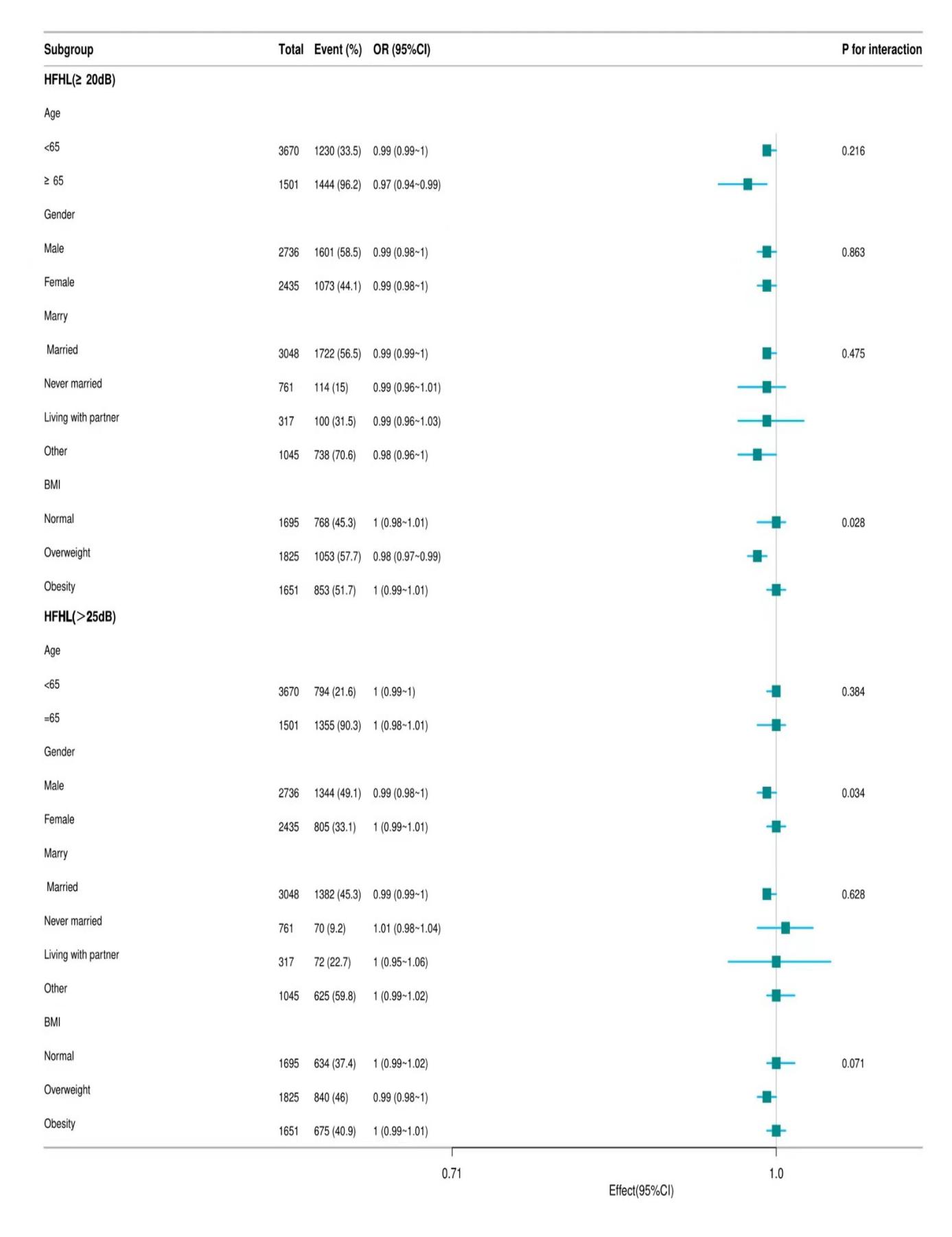

Supplement: Jiang and Chi supplementary material 1 — Jiang and Chi supplementary material [file S0022215125000635sup001.docx]
