# Supplementary material for: Association between HEI-2015 and hearing loss among American adults: National Health and Nutrition Examination Survey
Source: J Laryngol Otol. 2025 Sep;139(9):881–7. doi: 10.1017/S0022215125000635 (PMC12571590; doi:10.1017/S0022215125000635)
Supplement: Jiang and Chi supplementary material 2 — Jiang and Chi supplementary material [file S0022215125000635sup002.docx]

**Table 4, Table 5, Table 6 and Table 7**

**Table 4.** Association of covariates and low-frequency hearing loss.

| Variable | HL>25dB | | HL≥20dB | |  |
| --- | --- | --- | --- | --- | --- |
|  | OR_95CI | *P*_value | OR_95CI | *P*_value |  |
| Age | 1.1 (1.09~1.1) | <0.001 | 1.11 (1.1~1.12) | <0.001 |  |
| Female | 0.86 (0.76~0.99) | 0.032 | 0.87 (0.73~1.03) | 0.112 |  |
| Race |  |  |  |  |  |
| Other Hispanic | 1.56 (1.03~2.35) | 0.034 | 1.37 (0.81~2.32) | 0.237 |  |
| Non-Hispanic White | 2.04 (1.66~2.51) | <0.001 | 1.78 (1.37~2.31) | <0.001 |  |
| Non-Hispanic Black | 0.85 (0.64~1.12) | 0.244 | 0.73 (0.51~1.05) | 0.091 |  |
| Other Race-Including Multi-Racial | 0.86 (0.52~1.41) | 0.551 | 0.34 (0.13~0.85) | 0.021 |  |
| Marry |  |  |  |  |  |
| Never married | 0.19 (0.14~0.27) | <0.001 | 0.15 (0.09~0.26) | <0.001 |  |
| Living with partner | 0.43 (0.3~0.63) | <0.001 | 0.27 (0.15~0.51) | <0.001 |  |
| Other:widowed, divorced,or separated individuals | 2.28 (1.96~2.66) | <0.001 | 2.12 (1.76~2.55) | <0.001 |  |
| Family income | 0.88 (0.85~0.92) | <0.001 | 0.85 (0.81~0.9) | <0.001 |  |
| Education |  |  |  |  |  |
| 9-11th Grade (Includes 12th grade with no diploma) | 0.65 (0.5~0.85) | 0.001 | 0.61 (0.44~0.83) | 0.002 |  |
| High School Grad/GED or Equivalent | 0.58 (0.46~0.73) | <0.001 | 0.59 (0.45~0.77) | <0.001 |  |
| Some College or AA degree | 0.36 (0.29~0.46) | <0.001 | 0.34 (0.26~0.46) | <0.001 |  |
| College Graduate or above | 0.36 (0.29~0.46) | <0.001 | 0.33 (0.25~0.45) | <0.001 |  |
| Never smoke | 2.08 (1.79~2.41) | <0.001 | 1.89 (1.58~2.27) | <0.001 |  |
| Former smoke | 0.79 (0.65~0.96) | 0.019 | 0.56 (0.43~0.75) | <0.001 |  |
| Never drink | 1.35 (1.08~1.69) | 0.007 | 1.33 (1.01~1.75) | 0.041 |  |
| Former drink | 0.77 (0.62~0.94) | 0.012 | 0.86 (0.66~1.11) | 0.243 |  |
| Mild drink | 0.4 (0.3~0.52) | <0.001 | 0.41 (0.29~0.59) | <0.001 |  |
| Moderate drink | 0.32 (0.24~0.42) | <0.001 | 0.21 (0.14~0.32) | <0.001 |  |
| physical activity | 1 (1~1) | <0.001 | 1 (1~1) | <0.001 |  |
| Body mass index | 1 (0.99~1.01) | 0.987 | 0.99 (0.98~1.01) | 0.221 |  |
| CVD |  |  |  |  |  |
| Yes | 1 (reference) |  | 1 (reference) |  |  |
| No | 3.96 (3.31~4.74) | <0.001 | 3.68 (2.99~4.52) | <0.001 |  |
| Hypertension |  |  |  |  |  |
| Yes | 1 (reference) |  | 1 (reference) |  |  |
| No | 3.33 (2.9~3.83) | <0.001 | 3.52 (2.93~4.22) | <0.001 |  |
| DM |  |  |  |  |  |
| Yes | 1 (reference) |  | 1 (reference) |  |  |
| No | 2.57 (2.16~3.05) | <0.001 | 2.81 (2.29~3.44) | <0.001 |  |
| Noise |  |  |  |  |  |
| Yes | 1 (reference) |  | 1 (reference) |  |  |
| No | 1.6 (1.4~1.84) | <0.001 | 1.96 (1.65~2.33) | <0.001 |  |
| HEI-2015 | 1.02 (1.02~1.03) | <0.001 | 1.02 (1.01~1.03) | <0.001 |  |

with ≤25 dB HL as no hearing loss and >25 dB HL as having hearing loss, with <20 dB HL as no hearing loss and ≥20 dB HL as having hearing loss.OR: odds ratio; CI:confidence interval; Ref: reference;CVD:cardiovascular disease;HEI-2015:the Healthy Eating Index-2015.

**Table 5.** Association of covariates and speech-frequency hearing loss.

| Variable | HL＞25 | | HL≥20dB | |
| --- | --- | --- | --- | --- |
|  | OR_95CI | P-value | OR_95CI | P-value |
| Age | 1.11 (1.1~1.11) | <0.001 | 1.1 (1.09~1.11) | <0.001 |
| Female | 0.54 (0.47~0.61) | <0.001 | 0.53 (0.47~0.6) | <0.001 |
| Race |  |  |  |  |
| Other Hispanic | 1.14 (0.76~1.7) | 0.534 | 0.99 (0.69~1.41) | 0.943 |
| Non-Hispanic White | 1.86 (1.54~2.24) | <0.001 | 1.78 (1.51~2.09) | <0.001 |
| Non-Hispanic Black | 0.61 (0.47~0.8) | <0.001 | 0.67 (0.54~0.83) | <0.001 |
| Other Race-Including Multi-Racial  Marry | 0.77 (0.48~1.22) | 0.259 | 1.07 (0.74~1.53) | 0.731 |
| Never married | 0.14 (0.1~0.2) | <0.001 | 0.17 (0.13~0.22) | <0.001 |
| Living with partner | 0.44 (0.32~0.62) | <0.001 | 0.48 (0.37~0.63) | <0.001 |
| Other: widowed, divorced, or separated individuals | 1.9 (1.64~2.2) | <0.001 | 1.79 (1.56~2.07) | <0.001 |
| family income | 0.92 (0.88~0.96) | <0.001 | 0.94 (0.9~0.97) | <0.001 |
| Education |  |  |  |  |
| 9-11th Grade (Includes 12th grade with no diploma) | 0.62 (0.48~0.79) | <0.001 | 0.54 (0.43~0.69) | <0.001 |
| High School Grad/GED or Equivalent | 0.52 (0.41~0.64) | <0.001 | 0.53 (0.43~0.66) | <0.001 |
| Some College or AA degree | 0.33 (0.26~0.41) | <0.001 | 0.31 (0.25~0.39) | <0.001 |
| College Graduate or above | 0.34 (0.27~0.43) | <0.001 | 0.34 (0.27~0.43) | <0.001 |
| Never smoke | 2.45 (2.13~2.82) | <0.001 | 2.76 (2.42~3.15) | <0.001 |
| Former smoke | 0.77 (0.63~0.92) | 0.005 | 0.98 (0.84~1.14) | 0.792 |
| Never drink | 1.63 (1.31~2.03) | <0.001 | 1.87 (1.52~2.29) | <0.001 |
| Former drink | 1.01 (0.82~1.24) | 0.924 | 1.06 (0.88~1.28) | 0.538 |
| Mild drink | 0.47 (0.36~0.62) | <0.001 | 0.52 (0.41~0.65) | <0.001 |
| Moderate drink | 0.4 (0.3~0.51) | <0.001 | 0.47 (0.37~0.58) | <0.001 |
| physical activity | 1 (1~1) | <0.001 | 1 (1~1) | <0.001 |
| Body mass index | 1 (0.99~1.01) | 0.59 | 1 (0.99~1.01) | 0.927 |
| CVD |  |  |  |  |
| Yes | 1 (reference) |  | 1 (reference) |  |
| No | 4.34 (3.63~5.19) | <0.001 | 6.07 (4.98~7.4) | <0.001 |
| Hypertension |  |  |  |  |
| Yes | 1 (reference) |  | 1 (reference) |  |
| No | 3.43 (3~3.91) | <0.001 | 3.34 (2.97~3.76) | <0.001 |
| DM |  |  |  |  |
| Yes | 1 (reference) |  | 1 (reference) |  |
| No | 2.72 (2.3~3.21) | <0.001 | 3.07 (2.6~3.62) | <0.001 |
| Noise |  |  |  |  |
| Yes | 1 (reference) |  | 1 (reference) |  |
| No | 1.65 (1.45~1.88) | <0.001 | 1.61 (1.43~1.81) | <0.001 |
| HEI2015 score | 1.02 (1.02~1.03) | <0.001 | 1.03 (1.02~1.03) | <0.001 |

with ≤25 dB HL as no hearing loss and >25 dB HL as having hearing loss, with <20 dB HL as no hearing loss and ≥20 dB HL as having hearing loss.OR: odds ratio; CI:confidence interval; Ref: reference;CVD:cardiovascular disease;HEI-2015:the Healthy Eating Index-2015.

**Table 6.** Association of covariates and high-frequency hearing loss.

| Variable | HL≥20dB | | HL＞25 | |
| --- | --- | --- | --- | --- |
|  | OR_95CI | P_value | OR_95CI | P_value |
| Age | 1.13 (1.12~1.14) | <0.001 | 1.13 (1.13~1.14) | <0.001 |
| Female | 0.56 (0.5~0.62) | <0.001 | 0.51 (0.46~0.57) | <0.001 |
| Race |  |  |  |  |
| Other Hispanic | 0.86 (0.62~1.19) | 0.359 | 0.83 (0.58~1.19) | 0.313 |
| Non-Hispanic White | 1.61 (1.38~1.88) | <0.001 | 1.94 (1.66~2.28) | <0.001 |
| Non-Hispanic Black | 0.68 (0.56~0.82) | <0.001 | 0.71 (0.57~0.87) | 0.001 |
| Other Race-Including Multi-Racial | 0.8 (0.57~1.12) | 0.191 | 0.87 (0.6~1.25) | 0.456 |
| Marry |  |  |  |  |
| Never married | 0.14 (0.11~0.17) | <0.001 | 0.12 (0.09~0.16) | <0.001 |
| Living with partner | 0.35 (0.28~0.45) | <0.001 | 0.35 (0.27~0.47) | <0.001 |
| Other:widowed, divorced,or separated individuals | 1.85 (1.59~2.15) | <0.001 | 1.79 (1.56~2.07) | <0.001 |
| family income | 0.98 (0.95~1.01) | 0.213 | 0.97 (0.93~1) | 0.064 |
| Education |  |  |  |  |
| 9-11th Grade (Includes 12th grade with no diploma) | 0.45 (0.35~0.59) | <0.001 | 0.52 (0.41~0.67) | <0.001 |
| High School Grad/GED or Equivalent | 0.45 (0.36~0.57) | <0.001 | 0.52 (0.42~0.65) | <0.001 |
| Some College or AA degree | 0.27 (0.21~0.34) | <0.001 | 0.31 (0.25~0.39) | <0.001 |
| College Graduate or above | 0.34 (0.27~0.43) | <0.001 | 0.39 (0.31~0.49) | <0.001 |
| Never smoke | 3.05 (2.67~3.49) | <0.001 | 2.93 (2.57~3.35) | <0.001 |
| Former smoke | 0.96 (0.83~1.11) | 0.591 | 0.83 (0.72~0.97) | 0.02 |
| Never drink | 2.23 (1.81~2.75) | <0.001 | 1.88 (1.53~2.3) | <0.001 |
| Former drink | 1.19 (0.99~1.42) | 0.062 | 1.1 (0.92~1.32) | 0.301 |
| Mild drink | 0.57 (0.46~0.7) | <0.001 | 0.55 (0.44~0.68) | <0.001 |
| Moderate drink | 0.52 (0.42~0.64) | <0.001 | 0.4 (0.32~0.5) | <0.001 |
| physical activity | 1 (1~1) | <0.001 | 1 (1~1) | <0.001 |
| Body mass index | 1.01 (1~1.01) | 0.273 | 1 (0.99~1.01) | 0.476 |
| CVD |  |  |  |  |
| Yes | 1 (reference) |  | 1 (reference) |  |
| No | 7.66 (5.95~9.85) | <0.001 | 6.87 (5.55~8.5) | <0.001 |
| Hypertension |  |  |  |  |
| Yes | 1 (reference) |  | 1 (reference) |  |
| No | 4.41 (3.91~4.97) | <0.001 | 4.16 (3.69~4.67) | <0.001 |
| DM |  |  |  |  |
| Yes | 1 (reference) |  | 1 (reference) |  |
| No | 4.37 (3.59~5.31) | <0.001 | 3.77 (3.17~4.48) | <0.001 |
| Noise |  |  |  |  |
| Yes | 1 (reference) |  | 1 (reference) |  |
| No | 1.3 (1.15~1.46) | <0.001 | 1.48 (1.32~1.67) | <0.001 |
| HEI2015 score | 1.03 (1.02~1.03) | <0.001 | 1.03 (1.03~1.04) | <0.001 |

with ≤25 dB HL as no hearing loss and >25 dB HL as having hearing loss, with <20 dB HL as no hearing loss and ≥20 dB HL as having hearing loss.OR: odds ratio; CI:confidence interval; Ref: reference;CVD:cardiovascular disease;HEI-2015:the Healthy Eating Index-2015.

**Table 7.** Association between the Healthy Eating Index-15(HEI-2015) and hearing loss.

| Quintiles | OR (95% CI) | | | | | | | | |
| --- | --- | --- | --- | --- | --- | --- | --- | --- | --- |
|  | No. | Crude | P-value | Model 1 | P-value | Model 2 | P-value | Model 3 | P-value |
| HEI-2015(0-100) | Low-frequency hearing loss(≥20dB) |  |  |  |  |  |  |  |  |
| F(0-59) | 3627.0 | 1(Ref) |  | 1(Ref) |  | 1(Ref) |  | 1(Ref) |  |
| D(60-69) | 959.0 | 1.81 (1.53~2.13) | <0.001 | 1.01 (0.83~1.24) | 0.909 | 1.05 (0.86~1.29) | 0.624 | 1.06 (0.86~1.3) | 0.575 |
| C(70-79) | 446.0 | 1.87 (1.5~2.33) | <0.001 | 0.73 (0.55~0.95) | 0.021 | 0.77 (0.59~1.01) | 0.061 | 0.77 (0.58~1.01) | 0.058 |
| B(80-89) | 125.0 | 1.59 (1.06~2.39) | 0.024 | 0.5 (0.31~0.8) | 0.004 | 0.55 (0.34~0.89) | 0.015 | 0.54 (0.34~0.88) | 0.013 |
| A(90-100) | 14.0 | 1.78 (0.56~5.69) | 0.332 | 0.67 (0.17~2.59) | 0.558 | 0.71 (0.19~2.73) | 0.621 | 0.68 (0.18~2.62) | 0.578 |
| Trend test | 5171.0 |  | <0.001 |  | 0.002 |  | 0.015 |  | 0.014 |
| HEI-2015(0-100) | low-frequency hearing loss (>25 dB) |  |  |  |  |  |  |  |  |
| F(0-59) | 3627.0 | 1(Ref) |  | 1(Ref) |  | 1(Ref) |  | 1(Ref) |  |
| D(60-69) | 959.0 | 1.88 (1.53~2.3) | <0.001 | 1.01 (0.8~1.28) | 0.93 | 1.04 (0.82~1.32) | 0.758 | 1.04 (0.82~1.33) | 0.731 |
| C(70-79) | 446.0 | 1.82 (1.38~2.39) | <0.001 | 0.69 (0.5~0.94) | 0.021 | 0.71 (0.52~0.97) | 0.034 | 0.7 (0.51~0.96) | 0.029 |
| B(80-89) | 125.0 | 1.57 (0.94~2.61) | 0.086 | 0.53 (0.3~0.94) | 0.03 | 0.56 (0.32~1) | 0.048 | 0.54 (0.3~0.96) | 0.035 |
| A(90-100) | 14.0 | 1.55 (0.35~6.96) | 0.567 | 0.58 (0.11~2.96) | 0.509 | 0.6 (0.12~3.07) | 0.536 | 0.58 (0.11~3) | 0.516 |
| Trend test | 5171.0 |  | <0.001 |  | 0.006 |  | 0.014 |  | 0.011 |
| HEI-2015(0-100) | speech-frequency hearing loss (≥20 dB) |  |  |  |  |  |  |  |  |
| F(0-59) | 3627.0 | 1(Ref) |  | 1(Ref) |  | 1(Ref) |  | 1(Ref) |  |
| D(60-69) | 959.0 | 1.83 (1.58~2.11) | <0.001 | 1 (0.82~1.22) | 0.97 | 1.05 (0.86~1.28) | 0.642 | 1.06 (0.87~1.3) | 0.576 |
| C(70-79) | 446.0 | 1.77 (1.45~2.16) | <0.001 | 0.66 (0.5~0.87) | 0.003 | 0.71 (0.54~0.93) | 0.014 | 0.7 (0.53~0.93) | 0.013 |
| B(80-89) | 125.0 | 2.47 (1.72~3.54) | <0.001 | 0.88 (0.55~1.39) | 0.58 | 0.97 (0.61~1.54) | 0.892 | 0.98 (0.61~1.56) | 0.929 |
| A(90-100) | 14.0 | 0.8 (0.25~2.56) | 0.71 | 0.15 (0.04~0.64) | 0.01 | 0.16 (0.04~0.64) | 0.01 | 0.14 (0.03~0.56) | 0.005 |
| Trend test | 5171.0 |  | <0.001 |  | 0.009 |  | 0.047 |  | 0.044 |
| HEI-2015(0-100) | speech-frequency hearing loss (>25 dB) |  |  |  |  |  |  |  |  |
| F(0-59) | 3627.0 | 1(Ref) |  | 1(Ref) |  | 1(Ref) |  | 1(Ref) |  |
| D(60-69) | 959.0 | 1.73 (1.48~2.02) | <0.001 | 0.9 (0.73~1.11) | 0.324 | 0.93 (0.75~1.14) | 0.482 | 0.93 (0.75~1.15) | 0.507 |
| C(70-79) | 446.0 | 1.9 (1.54~2.35) | <0.001 | 0.74 (0.56~0.98) | 0.034 | 0.78 (0.59~1.03) | 0.076 | 0.78 (0.59~1.03) | 0.075 |
| B(80-89) | 125.0 | 2.19 (1.51~3.18) | <0.001 | 0.78 (0.49~1.24) | 0.296 | 0.85 (0.53~1.36) | 0.5 | 0.84 (0.52~1.33) | 0.451 |
| A(90-100) | 14.0 | 1.46 (0.46~4.65) | 0.527 | 0.4 (0.1~1.68) | 0.212 | 0.43 (0.1~1.78) | 0.246 | 0.43 (0.1~1.77) | 0.243 |
| Trend test | 5171.0 |  | <0.001 |  | 0.018 |  | 0.061 |  | 0.056 |
| HEI-2015(0-100) | high-frequency hearing (≥20 dB) |  |  |  |  |  |  |  |  |
| F(0-59) | 3627.0 | 1(Ref) |  | 1(Ref) |  | 1(Ref) |  | 1(Ref) |  |
| D(60-69) | 959.0 | 1.75 (1.52~2.03) | <0.001 | 0.74 (0.59~0.92) | 0.008 | 0.77 (0.61~0.96) | 0.022 | 0.76 (0.61~0.96) | 0.02 |
| C(70-79) | 446.0 | 2.07 (1.69~2.54) | <0.001 | 0.61 (0.44~0.85) | 0.003 | 0.66 (0.47~0.92) | 0.013 | 0.66 (0.47~0.91) | 0.012 |
| B(80-89) | 125.0 | 2.48 (1.69~3.65) | <0.001 | 0.5 (0.29~0.87) | 0.013 | 0.55 (0.32~0.95) | 0.031 | 0.52 (0.3~0.91) | 0.021 |
| A(90-100) | 14.0 | 2.03 (0.68~6.06) | 0.206 | 0.54 (0.09~3.36) | 0.506 | 0.54 (0.09~3.29) | 0.506 | 0.47 (0.08~2.77) | 0.406 |
| Trend test | 5171.0 |  | <0.001 |  | <0.001 |  | <0.001 |  | <0.001 |
| HEI-2015(0-100) | high-frequency hearing (>25 dB) |  |  |  |  |  |  |  |  |
| F(0-59) | 3627.0 | 1(Ref) |  | 1(Ref) |  | 1(Ref) |  | 1(Ref) |  |
| D(60-69) | 959.0 | 1.9 (1.65~2.19) | <0.001 | 0.9 (0.72~1.12) | 0.336 | 0.94 (0.75~1.18) | 0.576 | 0.94 (0.75~1.18) | 0.599 |
| C(70-79) | 446.0 | 2.34 (1.91~2.85) | <0.001 | 0.8 (0.58~1.09) | 0.162 | 0.84 (0.61~1.16) | 0.297 | 0.84 (0.61~1.16) | 0.292 |
| B(80-89) | 125.0 | 2.93 (2.03~4.24) | <0.001 | 0.81 (0.48~1.38) | 0.443 | 0.88 (0.52~1.5) | 0.651 | 0.86 (0.5~1.46) | 0.571 |
| A(90-100) | 14.0 | 3.18 (1.06~9.51) | 0.038 | 1.46 (0.23~9.16) | 0.685 | 1.47 (0.24~8.97) | 0.674 | 1.33 (0.22~7.84) | 0.754 |
| Trend test | 5171.0 |  | <0.001 |  | 0.143 |  | 0.347 |  | 0.308 |

Quintiles(A,B,C,D,F); OR: odds ratio; CI:confidence interval; Ref: reference. Model 1 was adjusted for sociodemographic variables (Age, Sex, Race, Marry, PIR, Education). Model 2 was adjusted for sociodemographic variables (Age, Sex, Race, Marry, PIR, Education), smoke, alcohol, physical activity, Body mass index. Model 3 was adjusted for sociodemographic variables (Age, Sex, Race, Marry, PIR, Education), smoke, alcohol, physical activity, Body mass index, CVD, hypertension, DM, noise
